# Supplementary material for: A whole-animal phenotypic drug screen identifies suppressors of atherogenic lipoproteins
Source: eLife. 2026 Aug 27;14:RP105314. doi: 10.7554/eLife.105314 (PMC13521566; doi:10.7554/eLife.105314)
Supplement: Supplementary file 2 [file elife-105314-code1.zip › JHDL_primary_result/Cytidine 5'-diphosphate┬átrisodium┬ásalt/Cytidine 5'-diphosphate┬átrisodium┬ásalt_JHDL_27_005_025_2019_11_08_11_10.pdf]

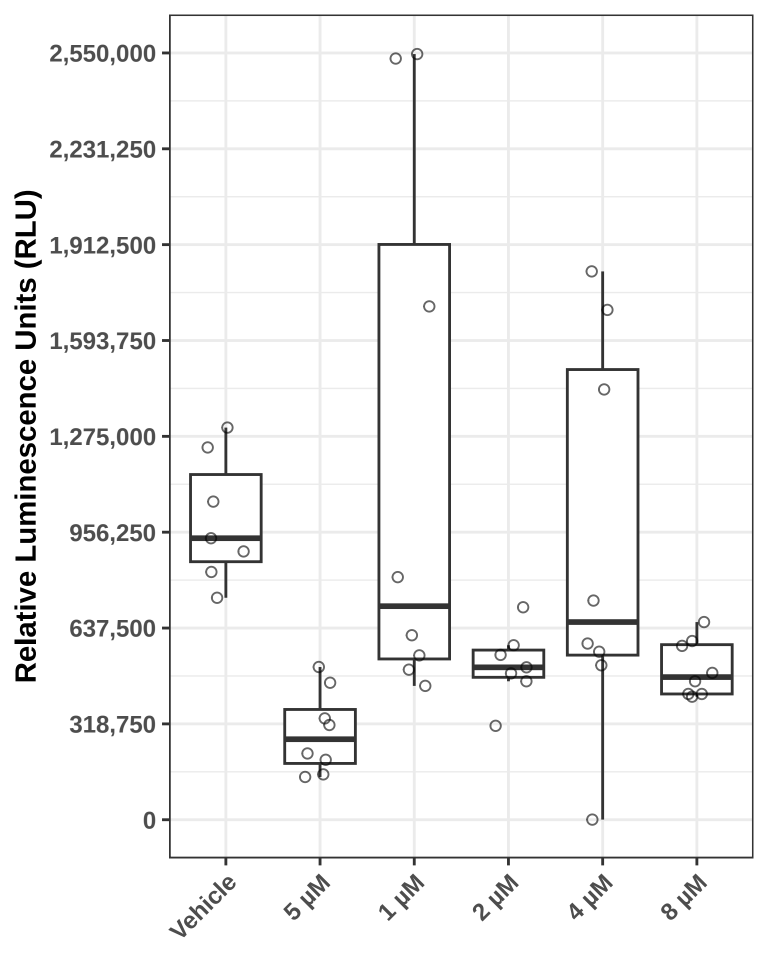
Relative Luminescence Units (RLU)
2,550,000
2,231,250
1,912,500
1,593,750
1,275,000
956,250
637,500
318,750
0
Vehicle
5 µM
1 µM
2 µM
4 µM
8 µM
